# Of primary health care reforms and pandemic responses: understanding perspectives of health system actors in Kerala before and during COVID-19

**DOI:** 10.1186/s12875-023-02000-0

**Published:** 2023-03-01

**Authors:** Hari Sankar D, Jaison Joseph, Gloria Benny, Devaki Nambiar

**Affiliations:** 1 The George Institute for Global Health, India, 308, Third Floor, Elegance Tower, Plot No. 8, Jasola District Centre, New Delhi, 110025 India; 2 Faculty of Medicine, University of New South Wales, Sydney, Australia; 3 Prasanna School of Public Health, Manipal Academy of Higher Education, Manipal, India

**Keywords:** Universal Health Coverage, Family Health Centres, Primary Care, Kerala, Aardram

## Abstract

**Background:**

In 2016, the Government of the southern Indian state of Kerala launched the Aardram mission, a set of reforms in the state’s health sector with the support of Local Self Governments (LSG). Primary Health Centres (PHCs) were slated for transformation into Family Health Centres (FHCs), with extended hours of operation as well as improved quality and range of services. With the COVID-19 pandemic emerging soon after their introduction, we studied the outcomes of the transformation from PHC to FHC and how they related to primary healthcare service delivery during COVID-19.

**Methods:**

A qualitative study was conducted using In-depth interviews with 80 health system actors (male *n* = 32, female *n* = 48) aged between 30–63 years in eight primary care facilities of four districts in Kerala from July to October 2021. Participants included LSG members, medical and public health staff, as well as community leaders. Questions about the need for primary healthcare reforms, their implementation, challenges, achievements, and the impact of COVID-19 on service delivery were asked. Written informed consent was obtained and interview transcripts – transliterated into English—were thematically analysed by a team of four researchers using ATLAS.ti 9 software.

**Results:**

LSG members and health staff felt that the PHC was an institution that guarantees preventive, promotive, and curative care to the poorest section of society and can help in reducing the high cost of care. Post-transformation to FHCs, improved timings, additional human resources, new services, fully functioning laboratories, and well stocked pharmacies were observed and linked to improved service utilization and reduced cost of care. Challenges of geographical access remained, along with concerns about the lack of attention to public health functions, and sustainability in low-revenue LSGs. COVID-19 pandemic restrictions disrupted promotive services, awareness sessions and outreach activities; newly introduced services were stopped, and outpatient numbers were reduced drastically. Essential health delivery and COVID-19 management increased the workload of health workers and LSG members, as the emphasis was placed on managing the COVID-19 pandemic and delivering essential health services.

**Conclusion:**

Most of the health system actors expressed their belief in and commitment to primary health care reforms and noted positive impacts on the clinical side with remaining challenges of access, outreach, and sustainability. COVID-19 reduced service coverage and utilisation, but motivated greater efforts on the part of both health workers and community representatives. Primary health care is a shared priority now, with a need for greater focus on systems strengthening, collaboration, and primary prevention.

**Supplementary Information:**

The online version contains supplementary material available at 10.1186/s12875-023-02000-0.

## Background

The Astana declaration of 2022 reaffirmed Primary Health Care (PHC) as the cornerstone for achieving Universal Health Coverage (UHC) and investments in improving the PHC system as the most efficient and inclusive approach to attain health-related United Nations Sustainable Development Goal 3 (SDG) by 2030 [1, 2]. Countries across the globe stand at varying stages on the UHC path: a modelling study from 67 Lower and Middle-Income Countries (LMICs) estimated that current PHC spending has to be at least doubled to make needed improvements in their systems and ensure PHC services are universally accessible [3]. Countries have committed to UHC and agreed to monitor their progress towards attaining it, even as there exist some critiques of the global consensus around what is to be prioritised in PHC and UHC reform [4].

Indian health policy reflects PHC and UHC as priorities as well: in 2015, the Ayushman Bharat-Pradhan Mantri Jan Arogya Yojana (AB-PMJAY) was rolled out by the Government of India with a component to provide universal access to PHC services to all its people [5]. Public health is a state subject in India and several Indian states had – before this central government reform, but also following it – devised tailor-made reforms to improve PHC service delivery [6–8].

The Kerala model of development is acclaimed for the investments in education, healthcare social infrastructure which yielded improved health outcomes like high life expectancy, low infant mortality and low birth rate [9]. Investment in the health sector has traditionally remained a priority area for governments in Kerala since the formation of the state [10]. In 2016, The Government of Kerala, through the Aardram mission [11], introduced a series of reforms in the health sector of the state with the support of Local Self Governments (LSGs). Primary Health Centres were slated for transformation into Family Health Centres (FHCs), with extended hours of operation as well as improved quality and range of services [11]. Kerala had early on sought to operationalise the constitutional obligation to decentralise power; as early as 1995 the state transferred funds, functions and functionaries of several government institutions including health to LSGs, meaning that two decades on, Primary Health Centres (now FHCs) roughly catering to 30,000 population in Kerala are managed by LSGs along with the health department. A typical PHC in Kerala has a one Medical Officer (MO) who provides clinical services and also supervises the public health team in the PHC. Every PHC has five to six subcentres roughly catering five thousand population. The public health team consists of a Health Inspector (HI) who supervises Junior Health Inspectors (JHI) in implementing communicable and non-communicable disease control activities. The Public Health Nurse (PHN) supervises a team of five to six Junior Public Health Nurses (JPHN) to provide a range of services to the population in their catchment area; they are supported by community health workers (CHW) named ASHAs, each of whom is assigned about a thousand individuals to support and connect with the health system [12]. LSGs have a controlling stake in PHCs and support them with additional human resources, maintenance funds, medicines and consumables [13]. The FHC program upgraded erstwhile resourcing, increasing the resources to three MOs and four staff nurses in FHCs, as well as the introduction of newer services at the primary level like Chronic Obstructive Pulmonary Disease management (COPD), diabetic retinopathy and depression screening [12, 14, 15]. The FHC program in Kerala under the Aardram Mission was the largest investment the state had made in recent years to improve health care infrastructure, with over 5,289 posts of hospital workers created and doubling the plan investment in the health sector [16].

The FHC program implementation was steadily progressing in the state when the first case of COVID - 19 in India was reported in Kerala in January 2020. Kerala's state enforced lockdown measures were followed by the national lockdown. Kerala’s COVID - 19 management efforts during the first wave of the pandemic received global acclaim. While case tallies in the state remained high, case fatality was low throughout, with early and high uptake of vaccination as well [17]. Some have credited this response to the existing strengths of the health system, particularly at the primary care level [18].

It could be said that the test of any reform is a moment of crisis. Aggregate figures used for monitoring mask the more in-depth nuances, stories, lessons and challenges that undergird success and failure. We contend that even as tracking of PHC reforms has been essential in understanding the status, qualitative studies which capture implementer perspectives and user accounts of health programs are vital in informing the policymakers about the barriers and enablers of the program locally [19]. Data on experiences /expertise collected through personal accounts are essential in course correction of program execution and generating future implementation plans. Kerala’s public health system, which is heavily supported by LSGs, presents a scenario which has multiple stakeholders other than health department personnel involved with implementing health reforms.

In the case of Kerala, there has been some work to monitor UHC-relevant PHC reforms [20, 21]. A 2021 health department report found that annual outpatient numbers in primary care institutions grew almost 24% between 2019–20 and 2017–18, reaching 31.5 million by 2020 post health reform [22]. An early evaluation in a single FHC with health staff and patients reported increased patient friendliness and improved service delivery [23]. Studies which evaluate the primary health reforms in Kerala specifically from the perspective of health system actors – from implementers- is lacking. Considering the system changes, as well as the shocks to the system introduced by COVID - 19 as well as floods and other disasters preceding it, we carried out a study to understand the outcomes of the FHC program from the perspectives of key supply-side actors before and during the context of COVID-19.

## Methods

This narrative research study constituted the qualitative component of a larger mixed methods health system research project in Kerala titled “Assessing equity of Universal Health Coverage in India: From data to decision-making using mixed methods” [24]. Study sites were identified through multistage random sampling; Kerala’s 14 districts were grouped into four categories using an index developed through principal component analysis of human development indicators from the National Family Health Survey (NFHS) Round 4 (2015–16) data [25]. One district was randomly chosen from each of the four groups, two primary care health facilities in each district were randomly selected from the district facility list. The details of sampling and selection criteria are detailed elsewhere [26]. We conducted In-Depth Interviews (IDI) with 80 health system actors across the eight primary care facilities/FHCs and four districts in Kerala from July to October 2021. We employed a purposive criterion sampling approach: study participants were selected from three categories from each study site using purposive criterion sampling where the intent was to include health system actors involved with FHC/PHC functioning from: i) Elected representatives of LSG (panchayat) (*n* = 17) who held positions of Panchayat President (PP), Vice president (VP) Health Standing Committee Chairperson (HSC) and ward members; ii)Health Care Providers (HCP) (*n* = 40) who were posted as Medical Officers (MO), Staff Nurses (SN), and field staff like HI JPHN, JHI, PHN, Palliative care Nurses (PN) in health facilities selected for study as well as iii) Community health workers (ASHA) and local community leaders(*n* = 23) working in the catchment area of selected facilities.

The study was approved by the Institutional Ethics Committee of The George Institute for Global Health (Project Number 05/2019). A three-member research team with qualitative research training consisting of two male research fellows and one female research assistant carried out the fieldwork, supervised by a senior health systems researcher. Administrative approval was taken from the Department of Health and Family Welfare, Government of Kerala. The team met the District Medical Officers (DMO) of four districts, shared the departmental permissions and outlined the study objectives and methods in detail and requested permission. After receiving permission from the DMO, permission from the MOs of each of the eight facilities as well as Panchayat presidents was sought, using the same process. HCPs who belonged to the identified categories and who agreed to participate in the study were briefed about the objectives of the study.

The pilot-test of the questionnaire was done with one MO and an HI from department of health, Kerala and an LSG member from Thiruvananthapuram district of Kerala. The feedback from piloting of questionnaire helped to refine the questionnaire, set the flow and pace of interview, remove redundancy, record interview time and improve the cue for interview questions. The semi structured questionnaire in English /Malayalam were shared with the participants whenever possible, before the interview. As the COVID -19 pandemic emerged as a major determinant in the study period specific questions were added to study its impact. The semi-structured questionnaire used in the study asked the health system actors their view on the need for primary health care reform, their role(s) in implementing this reform through the FHC upgradation process, challenges faced during implementation of the FHC program, outcomes of the program (including the impact of COVID-19 therein). Interview questions of the topic guide are attached in (Supplementary table 1) and were adapted from Mohindra and colleagues prior elicitation based qualitative research with populations facing disadvantage in Kerala [27]. HCPs were met in person at a time and place of their convenience for conducting the interviews. Medical officers and health inspectors were interviewed in their offices, while other HCPs were interviewed in common meeting room where privacy could be ensured (i.e. scheduling at a time when the room was not being used). Most of the IDIs were carried out in person, five HCPS who were unable to meet in person were interviewed through telephone. The interviews were conducted strictly adhering to COVID -19 protocols prescribed by the state government, i.e. interviewers and participants wore a mask all the time and physical distancing was maintained. The IDIs with LSG members were carried out first by meeting Panchayat Presidents and briefing them about the study. The elected LSG members were met with in person in the LSG office for conducting the IDIs. The interviews were conducted in official rooms of the Panchayat President, Vice President and Health Standing Committee members. A hard copy of the Participant Information Sheet (PIS) was handed to each participant for the in-person interviews and signed informed consent was taken for participating in the study and for recording the interviews. For interviews conducted online, PIS was sent over mail/Whatsapp and a signed soft copy was obtained. Before commencing the interview, the participants shared the duly signed consent form with the researchers. Interviews were conducted in Malayalam and lasted for 20–60 min. To obtain context and perspectives of health system actors in various capacities and geographies pertaining to each of the study sites across four districts the interviews with all the pre-set list of participants were completed even though achieving early data saturation was reached with some of the study topics. The response rate of the study was 96%, as three health system actors could not participate in the interview due to their busy schedules and after multiple failed attempts to schedule, we decided to remove them from the study. All the interviews were audio-recorded, and the recordings were secured in a password-protected database with researcher-only access. Detailed field notes of the interviews were written by the researchers to support the transcripts. Transcription was done by a professional transcription firm that transliterated the Malayalam interviews to transcripts in English. The quality checks of transcripts were done by the research team.

Inductive analysis of data was done using ATLAS.ti 9. Four members (DN,HS, JJ and GB) created a basic coding framework and developed a codebook iteratively. The codes that emerged were discussed and conflicts resolved. Codes were finalised and then applied by all coders across the dataset. The codes used for the study are described in Additional file 1. All coders’ ATLAS.ti files were merged, codes indexed and charted with emerging themes being discussed in weekly meetings over several months. The codes were arranged into themes and consolidated into a narrative summary and laid out in the results section.

## Results

### Participant characteristics

We conducted IDIs with 80 participants aged 30 to 63, of whom 60% (*n* = 48) were women (see Table 1). Out of the total participants recruited for the study, 30% were LSG members. The range of professional work experience ranged from first-time elected representatives who had been working for just six months to health care providers and community leaders with over thirty years of experience.

**Table 1:** Participant characteristics

Category	Designation	Professional experience range (in years)	Female	Male	Total
LSG members and community leaders	Panchayat President	6 months -5 years	3	4	7
	Panchayat Vice-President	5 years	0	1	1
	Health Standing Committee Member	6 months-10 years	3	5	8
	Ward Member	7 months	0	1	1
	Community Leader	10–30 years	1	6	7
Health Care Providers	Medical Officer	1–15 years	5	3	8
	Health Inspector	25–30 years	1	5	6
	Public Health Nurse	25–32 years	4	0	4
	Junior Health Inspector	14–26 years	0	7	7
	Junior Public Health Nurse (JPHN)	6–21 years	11	0	11
	Nursing Officer (NO)	1–6 years	3	0	3
	Palliative Nurse (PN)	8 years	1	0	1
	Community Health Worker (CHW)	10–13 years	16	0	16
	Total Participants		48	32	80

The years of experience served in the position was considered for the study. Most of the elected representatives had decades of political experience but years served in positions like president is indicated in the study

Our analysis yielded four themes, namely: the need for PHC reforms, the content of reforms, challenges encountered, and finally, impact of reforms including in relation to COVID-19.

- i) The need for primary health care reforms

Most participants felt that the PHC should be an institution that guarantees preventive, promotive, and curative care to the poorest section of society and can help in reducing the high cost of care. The recent reform to convert PHCs to FHCs was welcomed by many participants, as evidenced by a comment made by a frontline health worker in Kollam “It is the people's wellbeing that the government wants -… as a part of this, for the poor people, for people who are financially backwards to buy medicines, etc., these facilities are made.” (CHW KLM)

Other participants opined that the state bore responsibility for protecting the health and providing education to its people and that this commitment had been displayed by successive governments in Kerala. The present reform was seen as a continuation of this. A community leader from Thiruvananthapuram had this to say:

*If you look at the history of Kerala, irrespective of the ruling party- even if they differ in some manner- the state always had a strong public health policy, right from the first democratic government that came to power in 1957. Along with or instead of building multispecialty hospitals for curative medicine, a public health system was built in the state…, HIs and JPHN form a large network that works at the Panchayat level among the people….. through the “Aardram’ mission, the government has been able to increase public* participation in the health sector. (CL TVM)

A conflicting opinion about the investment in primary care through the recent reform was made by a HCP who felt the existing primary care system was robust enough and rather than investing more in primary care that the focus should be on secondary care.

*In a state like Kerala, there is no need to invest a huge amount in primary healthcare. In our state, most people are aware of the importance of such things. Before the Aardram Mission, we had NCD programs. We covered the maximum number of people in that, and they used to buy medicines from the PHCs. There are not many remote areas in Kerala. Therefore, I don't think it is a necessity. What I feel is that, if we use half of this fund for adding more facilities to Taluk Hospitals and hospitals above that, we could utilise it better. (MO KLM)*

- ii) What happened as part of the reforms?

Post transformation to an FHC, LSG members and HCPs reflected on significant, immediately visible changes like extended outpatient hours (9 am -6 pm) and additional doctors and nurses posted in FHCs. Participants mentioned new services introduced in the upgraded FHCs that linked earlier reforms like the generation and use of electronic health records (through the E-health program) [28] to provision of precheck services by staff nurses: the nurse would check patient vitals and enter these on the e-health platform in advance of the actual consultation with the MO). Participants also noted the introduction of speciality clinics: Chronic Obstructive Pulmonary Disease (COPD) management (SWAAS) clinic, depression screening clinic and screening for diabetic retinopathy. It was observed that reforms ensured patient amenities, fully stocked laboratories, and pharmacies in FHCs. A community leader commented about the improvements in medicine supply ensured by the health department

*Yes. Changes are there. There aren't any issues in the case of medicines. Earlier, the allotted medicines would not be enough. So doctors will demand more medicines. And then we(Hospital management committee) had to arrange supplementary medicines. This is how we used to manage hospital-related activities. Now it's not needed.(CL KSD)*

Health care providers felt that infrastructural changes and better staffing had increased the efficiency of the facility, quality of care and improved data reporting standards. This in turn helped facilities heighten their ambition for and in many cases achieve national-level quality standards prescribed for PHCs in India. Staff reported contributing to these efforts and felt proud of being part of the system as reflected in the opinion of a staff nurse in Thiruvananthapuram: “So after Aardram Mission came a thing … the NQAS [National Quality Assurance Standards]. ……We document all of these, and all of this will be analysed by NQAS, I was able to contribute a lot to this as well. I consider myself to be lucky to have contributed to this.” (JPHN TVM)

HCPs and LSG members expressed the opinion that post-FHC transformation, the outpatient visit numbers had gone up, and the coverage achieved by the facility had also increased. Participants felt that the program had a significant impact by delivering high-quality service and reducing out of pocket expenditure. A medical officer from Kasargode described this with an example.

*“One benefit of this is that out-of-pocket expenditure is very less now. Earlier when we used to shut down at 12:30 PM, if someone cut their hands(injury) they had to rely on private hospitals. If they go to a private hospital, they need to register first, and then they need to pay a doctor's fee and on top of that, they need to pay for any additional expenses, like an injection. We are treating them for Rs. 5 ($0.6) by keeping the hospital open until 6 PM instead of leaving them to spend Rs. 500($6.3) in a private hospital, for a patient, it is Rs. 495 ($6.2) saved when they choose to come here. (MO KSD)*

Another outcome of the reform many participants referred to was an increase in confidence among people and trust in services offered by the government. A medical officer in Alappuzha noted:

*More people are coming here now. Those who used to depend on private hospitals are gradually coming here. I cannot demand that everybody should visit here but if the ratio of people who used to rely on private and public clinics earlier were 60:40 respectively now it has come to 50:50 or the public sector is leading. More people are visiting the PHC for NCD treatments. (MO ALP)*

- iii) Challenges in implementing reforms

Notwithstanding these early gains, participants also reported that the reforms introduced newer positions like staff nurses and increased the number of medical officers, but overlooked other staff positions like field health staff, cleaning staff and clerical staff. While improvements in FHCs were appreciated, basic infrastructural and accessibility issues of frontline institutions like subcentres remained, as the following quotes from Junior Public Health Nurses in Alappuzha and Thiruvananthapuram demonstrate: “The sub-centre is situated at the backside of the Panchayat building. We do face issues in terms of transportation facilities. People can visit the place but vehicles cannot enter the area….. Only patients who can walk come here for treatments.” (JPHN ALP)

*Even though a lot of human resources have increased, those have only happened in the treatment section. There are still a lot of drawbacks on the preventive side. Concerning us, a JPHN or a JHI should be working for a population of 5,000. But currently, a JPHN or JHI is working with 12,000 to 15,000 people. There are a lot of drawbacks because of this, and there has been no growth in the preventive side as a result of Aardram. (JPHN TVM)*

LSG leaders also reported the geographical location of the PHC site and lack of public transport challenged access to care for all sections of the population in the facility catchment area. They also expressed concern over sustained support for reforms (as many of them are bankrolled by LSG) as not every LSG in Kerala has strong own revenue streams: a Panchayat president from Thiruvananthapuram voiced this concern:

*The government has directed the Panchayat to appoint a doctor, a nurse and a pharmacist. Their salaries should be given using the Panchayat fund itself. Our Panchayat has a meagre own fund. We are holding on because we come under the government's general purpose. We do not receive even 50% of the expenditure as revenue. In such a situation, we will not be able to provide the services of medical staff. This is a huge crisis. (PP TVM)*

Other kinds of challenges were also reported concerning roles. For example, Medical Officers were designated as the implementing officer of PHC reforms, but this required them to lead health teams with subordinates that had far more experience. Establishing lines of accountability, then, could be challenging at times, an MO from Kasargod reflected on this

*The real issue for a Medical Officer is not about the administration, it is the age. We face a lot of seniority problems. After we join, we are going to lead a group of subordinates like Junior Health Inspectors or Health Inspectors who are …. 50 to 56 [years old]. …they may not be following protocol. They just assume that it is a new boy. (MO KSD).*

- iv) Impact of COVID -19 on primary health care reforms

The restrictions introduced as part of the state’s COVID-19 response hampered reforms. Lockdown restrictions caused outpatient visit numbers to drop and field-level health activities to be held in abeyance, including school and institution-based activities. Even essential health services like immunisation, maternal and child health services, as well as Non-Communicable Disease (NCD) services, were briefly shut down. Attention was instead focused on testing and tracing among expatriates returning home as well as containment and vaccination activities. The COVID First-Line Treatment Centres (CFLTC) and Domiciliary Care Centres (DCC) started in LSGs were supported by PHC staff. This was seen by some HCPs as an unprecedented burden: “This level of strain is there, if you ask any JPHN who is about to retire, they will tell you that they never faced a period when there was this much risk and strain in their service” (JPHN TVM*).*

Lockdown restrictions were eased in phases and PHC services were adjusted according to the restrictions at each time. Attempts were made to maintain essential primary health services virtually and to ensure delivery of medicines at home through field health staff and volunteers. LSGs and HCPs worked together to train volunteer Rapid Response Team members (RRT) who delivered essential medicine to people based on instructions from the HCPs along with the field health staff.

*Firstly, as I mentioned, NCD medicines were supplied to different patients' houses from each ward through ASHA workers (CHW), RRT members and other healthcare staff. They provided medicines periodically every month from hospitals. Then we made sure that all the training and meetings were conducted online and not in person. These are the main changes we adopted during this COVID-19 crisis. (MO TVM)*

Following an initial period of disruption, therefore, attempts were made to re-activate the resources and procedures introduced by primary health reforms.

## Discussion

This study sought to understand the implementors' perspectives on the recent primary health reforms in Kerala. We learned that HCPs, LSG members and community leaders saw value and appropriateness in investment in PHC services by the government. Participants reflected on the improvements brought in by the reforms through upgradation of infrastructure, human resource and quality of service delivery however the lack of focus on preventive component of PHC services remains a challenge. The emergence of COVID-19 pandemic disrupted the reforms but the adaptive measures by the system were introduced to ensure uninterrupted essential primary care health care delivery.

The health system actors we interviewed—elected leaders of LSG and HCPs—perceived the primary care reforms as a step in the right direction and defined PHC services as people-centred with preventive and promotive care components and many considered healthcare provision as a responsibility of the government. Alignment between local political leaders, decision makers and health providers about the need and scope of services is crucial to the success of any program. The WHO operational framework for PHC care describes the importance of commitment by political leaders in implementing PHC as multisectoral coordination for improving social, economic, environmental and commercial determinants of health is possible only with committed political leadership [29]. LSG leadership in Kerala appears to have the capacity and resources to carry out this stewardship role. This understanding can be attributed to the already existing robust PHC network in the state and over twenty-five years of decentralized governance, such that LSG roles in the health sector are actually in place at the grass-root level [30]. A review on decentralization and its health system impact(s) stated that role clarity, knowledge of local context and local decision-making can be critical determinants to the success of a program [31].

As reflected by our participants, a significant characteristic of the reform was introducing speciality clinics in FHCs for COPD and depression screening. SWAAS clinic was the first program in India to address the burden of COPD and Asthma through primary health care. as of January 2021 the program is reported to have screened over 148,870 patients, diagnosed nearly nineteen thousand COPD cases, and provided free medicines including inhalers [32, 33]. The depression screening program in FHCs has, as of July 2022, screened over sixty five thousand people and diagnosed nearly twelve thousand cases in the state [34]. Beyond this, our study suggests that FHC program may have escalated quality improvement activities in government health facilities of the state: 85 out of 932 primary care facilities in the state were certified by NQAS in 2021 [35]; the numbers continue to grow. A study to identify barriers and enablers to NQAS certification of the facilities in Kerala reported that transformation to FHCs ensured fully stocked pharmacies, diagnostics, patient amenities and improved commitment of the staff which is consistent with the reflections of our study participants [36].

Another study conducted among government doctors in Malappuram district in 2014, before the implementation of the FHC program, reported short patient interaction time in OPD and administrative work as challenges of working in the government health system [37] In 2021, post-FHC transformation, the MOs interviewed in our study reported that with additional doctors posted, efficiency had increased as there could be the division of administrative and clinical duties. A follow-up study exploring this difference using matching indicators could shed more light on this. Trust in government machinery and elected representatives has traditionally been high in Kerala [38] and with the state government's efforts in managing the COVID-19 pandemic, the public trust has only further increased. Our findings are echoed in other studies that have also concluded that people feel more confident to use in government health system post Aardram Mission and the COVID-19 pandemic in Kerala, the COVID-19 pandemic had an impact on public trust in the government health system globally [39–42].

The central government model of primary care reform, the Health and Wellness Centre (HWC) program focuses on developing subcentres (each PHC has at least six subcentres where outreach health services like immunization, health education etc. are delivered) with Middle-level Health providers (a trained nurse or AYUSH doctor) [43]. In contrast, till 2020, the FHC model was focused on PHC transformation, with an emphasis on posting additional MBBS doctors. Disease surveillance, health promotion, and palliative home care are all managed by public health staff in an FHC, but in the reform, no additional field health staff positions were created or posted; this was in fact identified by HCPs as a challenge in implementing the FHC program. The design of the FHC program itself was aimed at improving infrastructure and posting additional doctors and nurses for medical management [44]. Our study found that field health workers were dissatisfied with the lack of focus on developing subcentre infrastructure and field health service delivery through the FHC program. Global evidence from LMICs suggests that field-level public health activities that improve population health and non-physician health workers are a critical component of primary health care delivery [45, 46]. The Kerala Department of Health and Family Welfare has now started to integrate the HWC program into FHC by recruiting staff nurses as MLHP and improving the infrastructure of one subcentre in each FHC [47]. This is likely to directly address the concerns of frontline workers; further study can reveal the acceptability of these reforms for both supply and demand side actors as well as their impact(s) on population health.

LSG members and community leaders who participated in our study reflected that physical access to FHC remained a challenge for many in the community and this affects the utilization of services too. Kerala from as early as the nineties is reported to have a good network of roads and 78% of villages have health facilities available within five kilometres. A study in Kasaragod - one of the underdeveloped districts in the states-  also reported that the median distance to a public health facility was 6 km [48, 49]. Notwithstanding these improvements, transport and physical access were still seen as access barriers, particularly since already existing PHCs (which may have been hard to reach) were the ones chosen for FHC transformation: existing geographical challenges reportedly remained.

The role of decentralization in helping Kerala in improving population health is well discussed and documented [50, 51]. The ability of the local population to participate in health planning and the autonomy of LSGs to design and fund locally relevant interventions has produced successful models like palliative care cascade in the state [52]. In 2016, when the Aardram mission was launched, LSGs were a very important partner in implementation, particularly given the role they played in co-funding human resources and infrastructure upgradation. Our study results indicate that going forward, there could be a challenge with this as many LSGs were facing fund shortages, especially post COVID - 19. This raises an issue with the sustainability of the FHC model in which staff salaries are met through LSG funding.

The COVID - 19 pandemic and the restrictions placed as part of controlling the pandemic affected the PHC service delivery globally [53, 54] as in Kerala’s newly introduced FHC program. However, insofar as the FHC program itself was built on a strong edifice of reforms, backed by community participation and political will, the state had some early success in managing the pandemic [55]. Sustaining the reforms, managing (raised?) expectations of the public and continuing coordination and collaboration in non-emergency contexts while also expanding focus on the field and preventive care will be major areas of attention and concern going forward. Implementer perspectives are a useful way to understand supply side experiences and operational challenges related to the FHC program. Further research needs to be focused on the impact of disruptions in primary health service delivery during the COVID - 19 pandemic on population level service utilisation, perception of services, health outcomes, and knock on impacts (for example on livelihoods and household expenditure). There are likely impacts COVID - 19 has had on the design and functioning of the FHC program -whether short or long term, which also warrant examination. The progress made in improving FHC infrastructure and human resources is appreciable, but the study calls for policy action focusing on outreach health infrastructure and service delivery. A policy intervention for equipping the staff performing duties based on the original design principles of FHC is also required.

### Strengths and limitations

Our study captured and summarised the opinions of different health system actors including health care staff, community members and elected representatives providing a comprehensive information about the rollout of primary care reform in Kerala and how did it function during the COVID - 19 pandemic. There are currently very few studies that studied about the Family Health Centre model of Kerala, particularly in the context of COVID - 19 and it is this gap that our analysis helps fill. Notwithstanding this needed focus on an implementer lens – this analysis lacked perspectives of communities. This is a limitation as user perspectives are crucial in understanding any program. These perspectives are in the process of being gathered in the next stage of our project. Moreover, the timing of our data collection was after the second wave of COVID - 19 in Kerala: health workers were overwhelmed with vaccination duties, this may have affected their opinion and reflections about the FHC program which started three years before in 2018 and many components of the program being on hold for long period after. Ongoing research with supply and demand side actors, through a prospective mixed methods program of health systems research could continue to shed light on this.

## Conclusion

Supply-side actors involved with primary health care reforms in the southern Indian state of Kerala had clarity in the concept of what primary health care is and what their roles are. The FHC program improved infrastructure, but the creation of new posts as part of the program was skewed towards clinical roles. Physical access to facilities remained a barrier. COVID - 19 affected the implementation of the FHC program through essential PHC service delivery remains uninterrupted. The focus now should be to get the program back on track and think of a sustainable way ahead after a considerable time spent by the health system focusing exclusively on COVID - 19 management.

## Supplementary Information

**Additional file 1:** **Supplementary table 1.** Interview questions in topic guide and codes used for analysis.

## Data Availability

All datasets used for supporting the conclusions of this paper are available from the corresponding author on request.

## Abbreviations

FHC: Family Health Centre
PHC: Primary Health Centre
SDG: Sustainable Development Goal
LSGs: Local Self Governments
OOPE: Out of Pocket Expenditure
HWC: Health and Well ness Centres
ASHA: Accredited Social Health Activist
