# Supplementary material for: Of primary health care reforms and pandemic responses: understanding perspectives of health system actors in Kerala before and during COVID-19
Source: BMC Prim Care. 2023 Mar 1;24:59. doi: 10.1186/s12875-023-02000-0 (PMC9975828; doi:10.1186/s12875-023-02000-0)
Supplement: Supplementary file 1 — Additional file 1: Supplementary table 1. Interview questions in topic guide and codes used for analysis. [file 12875_2023_2000_MOESM1_ESM.docx]

| **Questions in the interview schedule** | **Codes** |
| --- | --- |
| Why do you think these health reforms were introduced? | PHCR_needoverall  PHCR_healthpromotion  PHCR_CP |
| Do you think that programs like Family Health Centre had brought a change in people’s lives? | PHCR_PHCtoFHC  PHCR_outcome  PHCR_opinion |
| Describe the administrative challenges if any, faced during implementation of the program like lack of human resource or funds etc?  What according to you were missing in the scope of health reforms in the past? | PHCR_Implchallenge  PHCR_Implchallenge_geography  PHCR_Implchallenge_infra  PHCR_Implchallenge_HR  PHCR_FHCvsSHC  PHCR_Whatismissing |
| What was the most affected health service in your institution(area) during COVID 19 lockdown and unlock period?  How did you manage to provide health services to needy people during the lockdown?  In your role what were the major duties you were involved in last year related to Covid 19 management ? (Jan- Dec 2020 ) COVID 19 pandemic | COVID_disrupt  COVID_additionalrole  COVID_burden  COVID_servicedesignchange |

**Supplementary table 1. Interview questions in topic guide and codes used for analysis**
